# Assessing the Effect of Cellulose Nanocrystal Content on the Biodegradation Kinetics of Multiscale Polylactic Acid Composites under Controlled Thermophilic Composting Conditions

**DOI:** 10.3390/polym15143093

**Published:** 2023-07-19

**Authors:** Priscila Esther Colli-Gongora, Nora Magally Moo-Tun, Pedro Jesús Herrera-Franco, Alex Valadez-Gonzalez

**Affiliations:** 1 Centro de Investigación Científica de Yucatán, A.C., Unidad de Materiales, Calle 43 # 130 Entre 32 y 34, Col. Chuburná de Hidalgo, Mérida C.P. 97205, Yucatán, Mexico; priscila.collig@gmail.com (P.E.C.-G.); pherrera@cicy.mx (P.J.H.-F.); 2 MTGREEN LAB, Calle 127 # 566 Col. San Antonio Xluch, Mérida C.P. 97205, Yucatán, Mexico; noram_22@hotmail.com

**Keywords:** multiscale biocomposites, PLA, cellulose nanocrystals, cellulose microfibers, biodegradation kinetics, controlled composting

## Abstract

This work studied the effect of cellulose nanocrystal (NCC) content on the biodegradation kinetics of PLA-based multiscale cellulosic biocomposites (PLAMCBs). To facilitate biodegradation, the materials were subjected to thermo-oxidation before composting. Biodegradation was carried out for 180 days under controlled thermophilic composting conditions according to the ASTM D 5338 standard. A first-order model based on Monod’s kinetics under limiting substrate conditions was used to study the effect of cellulose nanocrystal (NCC) content on the biodegradation kinetics of multiscale composite materials. It was found that thermo-oxidation at 70 °C for 160 h increased the biodegradability of PLA. Also, it was found that the incorporation of cellulosic fibrous reinforcements increased the biodegradability of PLA by promoting hydrolysis during the first stage of composting. Likewise, it was found that partial substitution of micro cellulose (MFC) by cellulose nanocrystals (NCCs) increased the biodegradability of the biocomposite. This increase was more evident as the NCC content increased, which was attributed to the fact that the incorporation of cellulose nanocrystals facilitated the entry of water into the material and therefore promoted the hydrolytic degradation of the most recalcitrant fraction of PLA from the bulk and not only by surface erosion.

## 1. Introduction

Polylactic acid (PLA) is a linear aliphatic polyester made from renewable resources that microorganisms in the environment can break down [1,2,3]. Multi-scale biodegradable materials using micro- and nano-sized inclusions are being developed to increase their performance and replace non-biodegradable synthetic plastics in dynamic and demanding sectors such as packaging, construction and the automotive industry [4,5,6]. Various research groups have developed multi-scale biocomposites with cellulosic reinforcements to improve their performance under high demands while maintaining biodegradability [7,8,9,10,11].

Several reports in the literature have studied the biodegradability of PLA [12,13,14,15,16] and its biocomposites with cellulose [17,18,19,20] or nanocellulose [15,21,22,23]. PLA composting is a method of disposing of PLA waste through microbial degradation in a controlled environment. The process involves breaking down PLA into water-soluble low-weight oligomers or into lactic acid, which can be used as a nutrient source by microorganisms during the composting process. Composting PLA has several advantages, including reducing waste in landfills, producing nutrient-rich soil amendments and reducing carbon emissions associated with traditional waste disposal methods. Recent studies have shown that abiotic hydrolysis is the rate-limiting step of PLA biodegradation under composting conditions [24,25,26,27]. Due to PLA hydrolysis being accelerated at temperatures above its glass transition temperature [28,29,30], PLA biodegradation proceeds easily during the thermophilic phase of the composting process, characterized by relatively high temperature (65–70 °C) [31,32,33]. Several researchers have studied the composting kinetics that governs the biodegradation of various types of waste, including biodegradable polymers, since this knowledge is valuable for properly operating industrial composting facilities.

Komilis [34] developed a first-order mathematical model based on Monod kinetics under limiting substrate conditions to describe the carbon organic degradation from vegetable waste to carbon dioxide under composting conditions. The model assumes that the total initial solid organic carbon of a solid waste consists, according to their hydrolysis facility, of three different fractions: readily (Csr), moderately (Csm) and slowly or recalcitrant (Css). Also, the model considers the hydrolysis of solid carbon to be the rate-limiting step of biodegradation during composting. Once hydrolyzed, the carbon fractions become undistinguishable intermediate aqueous carbon (Caq) mineralized by microorganisms. This model successfully studied the composting kinetics of six solid municipal solid wastes.

On the other hand, it is generally accepted that the complete degradation of biodegradable polymers during composting consists of two stages in series. In the first stage, the material undergoes hydrolysis, which can be both chemical (abiotic) and enzymatic (biotic), which favors the formation of low-molecular-weight oligomers or monomeric units that are soluble in water and therefore can be mineralized by the microorganisms present in the compost and converted into CO_2_ and water in a second stage [32].

Leejarkpai et al. [35] studied biodegradation under controlled composting conditions of biodegradable polymeric materials. These authors proposed that the degradation of the organic carbon present in these materials followed a degradation mechanism similar to that proposed by Komilis [34], that is, a first stage of hydrolysis, assuming the presence of three types of solid organic carbon based on its ease of hydrolysis, that is, easily, moderately and slowly hydrolyzable carbon that is subsequently mineralized by microorganisms in a second stage. In addition, these authors modified the Komilis mathematical model by including a lag phase, which frequently occurs in the composting of biodegradable polymers. Stloukal et al. [36] studied the biodegradation kinetics of PLA–montmorillonite (PLA/MMT) nanocomposites under controlled composting conditions using the Leejarkpai et al. [35] model. The mathematical model was able to adequately reproduce the release of CO_2_ from the nanocomposites prepared with pristine and organically modified MMTs, at a single concentration (5% wt/wt). Their results show that organically modified MMTs increase the rate and degree of biodegradation of pristine PLA. Likewise, Stloukal et al. [37] studied the effect of carbodiimide content on PLA biodegradation kinetics under controlled composting conditions using the Leejarkpai et al. [35] model. These authors also carried out the hydrolysis of PLA to compare with composting behavior. To do this, the mineralization term was eliminated from the mathematical model. Their results show that the hydrolysis stage of the solid fraction of organic carbon is very important in the biodegradation of PLA and that the incorporation of carbodiimide is capable of reducing the abiotic hydrolysis as long as a minimum amount of the additive is added. Once again, the mathematical model was able to adequately reproduce the release of CO_2_ from both abiotic hydrolysis and biodegradation in composting.

Sable et al. [38,39,40] in a series of studies have successfully used the Leejarkpai et al. [35] model to study the biodegradation kinetics of different polypropylene (PP)-based composite materials under controlled composting conditions. They have studied blends of PP with PLA and nanoclays with and without the presence of oxo-prodegradant additives. They also studied the effect of the grafting content of acrylic acid (0–35 wt%) on the PP. They recently reported the study of the effect of an abiotic pretreatment (UV accelerated aging) and the content of an oxo-prodegradant additive based on cobalt stearate on the PP biodegradation kinetics.

Similarly, Kalita et al. [41] have successfully studied the kinetics of biodegradation, under controlled composting conditions, of PLA and its blends with polyethylene (PE), microcrystalline cellulose (MCC), chitosan, cellulose nanocrystals (NCCs) and gum arabic using the Leejarkpai et al. [35] model.

However, to the best of our knowledge, biodegradation studies, under controlled composting conditions, of PLA-based multiscale composites containing micro- and nano-sized cellulosic fibrous reinforcements are scarce. So, there is still a lack of knowledge of these cellulosic reinforcements’ roles in PLA compostability.

This work studied the effect of cellulose nanocrystal content on the biodegradation kinetics of PLA-based multiscale cellulosic biocomposites. For this, PLA was blended with a combination of microcellulose (MFC) and cellulose nanocrystal (NCC) fibers while keeping the total content of cellulosic fiber reinforcements constant. The biocomposites were composted for 180 days, and the evolution of the CO_2_ was measured at certain time intervals using a respirometric system according to ASTM standard D 5338 [42]. To study the effect of NCC content on the biodegradation kinetics of these PLA-based multiscale biocomposites, the Leejarkpai et al. [35] model was used.

## 2. Materials and Methods

### 2.1. Materials

PLA Ingeo 3251D, with a number average molecular weight (Mn) of 47.4 kg/mol, a glass transition temperature of 57 °C and a melting point of 180 °C, was purchased from NatureWorks^®^ LLC (Minnetonka, MN, USA). Cellulose microfibers, with an average length of 500 ± 35 µm, diameter of 12 ± 2 µm and average aspect ratio of L/D = 40 were used. They were obtained from Henequen (*agave fourcroydes*) fibers according to the method described by Cazaurang et al. [43] and conditioned according to the method described in [44]. The lyophilized cellulose nanocrystals were acquired from the University of MAINE, with 0.94% by weight of sulfur on a dry basis and were used as received. They were produced by acid hydrolysis of the wood pulp with an H_2_SO_4_ solution (64% w/w) and had an average length of 296 ± 10 nm, an average diameter of 21.4 ± 2 nm and an average aspect ratio (L/D) of 14 [45]. The maleic anhydride powder 95% from Sigma-Aldrich was used as the coupling agent. For the study of biodegradation, mature compost from CICY A.C “Unidad de Recursos Naturales” was used.

### 2.2. Methods

#### 2.2.1. Biocomposite Preparation and Characterization

The different PLA-based multiscale biocomposite (PLAMCB) materials, described in Table 1, were obtained using a BRABENDER^TM^ high-sear mixing chamber. Maleic anhydride was used as a coupling agent in each of the composite materials, which were 0.5% of the total cellulose content (20%).

#### 2.2.2. PLAMCB Preparation

The biocomposites were prepared according to the methodology reported in [8]. Briefly, the PLA, MFC and the cellulose NCCs were dried under vacuum for 24 h at 50 °C before being used. A Brabender^TM^ high-shear mixing chamber with three heating zones was used, 170 °C being the temperature for each zone. Mixing time was 10 min at 50 rpm. The PLA was allowed to melt, and the cellulosic fibers and the coupling agent were subsequently added. Subsequently, the mixture was allowed to cool and stored in a desiccator at 25 °C with a relative humidity of 25%.

#### 2.2.3. Preparation of PLAMCB Specimens

Compression molding of the laminates was carried out at a temperature of 170 °C using 12 × 12 cm and 0.1 cm thick mold. Release wax was used to facilitate the demolding. Once the material was melted, the pressure was applied as follows: 2000 pounds for 1 min, 3500 pounds for 1 min and finally 5500 pounds for 5 min. Subsequently, the plates of the press were cooled with water, and the laminates were stored in a desiccator at room temperature and relative humidity of 50%. From the laminates, specimens of dimensions 0.5 × 0.5 × 0.1 cm were obtained. The specimens were dried at 50 °C under vacuum until constant weight was reached and stored in glass desiccators at 25 ± 1 °C and 25 ± 3 RH (%).

#### 2.2.4. Thermo-Oxidation Treatment

When PLA is exposed to heat and oxygen, a thermo-oxidation process occurs, leading to molecular weight drop and the formation of new carbonyl and hydroxyl groups [46]. For the thermo-oxidation of the PLAMCB samples, each of the formulations was placed separately in aluminum trays, and they were introduced to a convection oven for 160 h at 70 °C, after which time they were placed in a desiccator for 24 h to condition at room temperature.

#### 2.2.5. PLAMCB Spectroscopic and Thermal Characterization

##### Raman Spectroscopy

The Raman spectra of thermo-oxidized were collected between 3200–100 cm^−1^ using an inVia Renishaw Raman spectrometer (Renishaw, Gloucestershire, UK). A 532 nm laser at 5% power was used as the excitation radiation source. The exposure time was 100 ms.

##### Thermogravimetric Analysis (TGA)

The thermal behavior analysis of each material (10 mg) was carried out in a TGA 7 Perkin Elmer Thermogravimetric Analyzer in the temperature range of 40–600 °C at a heating rate of 10 °C/min and nitrogen flow of 100 mL/min in a nitrogen environment. The thermograms were recorded in triplicate, and the results reported are the average of the three measurements.

#### 2.2.6. Biodegradation under Controlled Composting Conditions

Specimens of 0.5 × 0.5 × 0.1 cm were placed directly in the compost and then incubated for six months according to ASTM D-5338 standard “Determining Aerobic Biodegradation of Plastic Materials under Controlled Composting Conditions”. The samplings were carried out every eight days. The compost used was purchased from CICY A.C.

#### 2.2.7. Compost Characterization

The characterization of the compost was carried out by determining the pH, dry matter by drying at 105 °C for 12 h and organic matter (OM) content by loss on ignition at 430 °C for 24 h. Total nitrogen (TN) and total organic carbon (TOC) were determined by elemental analysis using a Thermo-Scientific Flash 2000 Elemental Analyzer.

#### 2.2.8. Biodegradation System

**Figure 1:**
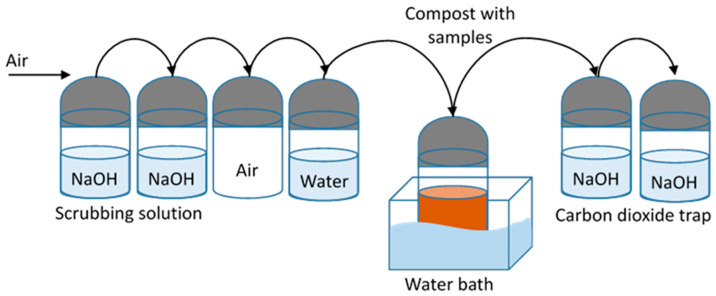
Composting experimental setup.

The experiments were carried out following ASTM D 5338 to determine the time and amount of CO_2_ released by the samples during the composting process (See Figure 1).

The system was located in a dark environment. The methodology was as follows: The air supplied by the compressor passed through a system of three containers; the first contained silica and the others NaOH, which cleaned the air of CO_2_ and H_2_O.

The CO_2_ and H_2_O free air passed through a piping system which supplied air to the composting bins that held the compost and sample. Composting containers were immersed in an aqueous medium at a controlled temperature of 65 °C.

In the composting containers, 50 g of compost and 5 g of the sample were placed, and the containers were weighed before starting the test. A control was carried out to monitor the effectiveness of the compost, which consisted of introducing a container that only contained compost. The positive control consisted of compost and starch and the negative control compost and polyethylene. The gases released by the biodegradation process passed through a pipe to a system consisting of three containers, of which one cleaned H_2_O and the other two trapped the CO_2_ released by the biodegradation process. The CO_2_ trapped in the last two bottles was titrated to determine the amount of CO_2_ released by the samples every eight days. 

#### 2.2.9. Biodegradation Study

Compostability was assessed using the ASTM Standard Test Method D 5338 [42]. These experiments were used to determine the rate and extent of biodegradation of plastic materials in a controlled and reproducible test environment. Biodegradability was measured as the percentage of the carbon from the test material that was mineralized to carbon dioxide during the test period. The CO_2_ generated during the biodegradation test was captured in 80 mL of 0.030 N NaOH and precipitated as NaCO_3_. The amount of CO_2_ produced was determined by titrating the NaOH in each trap with 0.025 N HCl, using phenolphthalein as an indicator.

The total amount of CO_2_ evolution was calculated with reference to the control reactor that did not have a sample, which was our blank (blank reactor). The percentage of biodegradation of the sample was calculated according to Equation (1):

(1) $$\%Biodegradation=\frac{{\left(CO_{2}\right)}_{T}-{\left(CO_{2}\right)}_{B}}{TCO_{2}}\times 100$$

where (CO_2_)_T_ is the accumulated amount of carbon dioxide that is generated in each reactor containing sample and compost over time, in grams per reactor, and (CO_2_)_B_ is the mean cumulative amount of evolved carbon dioxide in the blank reactor (only compost) in grams per reactor. The theoretical amount of CO_2_ (TCO_2_), in grams per reactor, was calculated using the following Equation (2):

(2) $$TCO_{2}=M_{Tot}*\;C_{Tot}*\frac{44}{12}$$

where TCO_2_ is the theoretical amount of carbon dioxide that the sample can produce in grams per reactor, M_Tot_ is the total dry solids in grams in the sample added to the reactors at the beginning of the test and C_Tot_, is the total proportion of organic carbon in the dry solids in the sample and was determined using a Thermo-Scientific Flash 2000 Elemental Analyzer. The amount of CO_2_ released during the biodegradation of the first samples was determined by titration of the NaOH solution of the test of each sample and the blank with hydrochloric acid 0.05 N to the phenolphthalein end point. The titration test was performed in triplicate with a frequency of 8 days during the whole incubation time. Once the biodegradation stage finished, the compost was characterized in terms of its pH, organic matter content, total carbon and nitrogen content.

### 2.3. Kinetics Analysis

Biodegradation of PLA proceeds via a two-stage mechanism. In the first step, hydrolysis of ester linkage occurs. This step can be accelerated by acid or bases and is affected by both temperature and moisture levels. In the primary degradation phase, no microorganisms are involved. As the average molecular weight diminishes, microorganisms present in the soil begin to digest the lower molecular weight lactic acid oligomers, producing carbon dioxide and water. PLA degrades rapidly in the composting atmosphere of high humidity and temperature (55–70 °C). But, at lower temperatures and/or lower humidity, the storage stability of PLA products is considered to be acceptable.

To find out the effect of the NCC content on the PLAMCBs biodegradation kinetics under controlled composting conditions, the Leejarkpai et al. [35] approach was used. The rate equations involved in the entire mechanism are as follows:

(3) $$\frac{dC_{r}}{dt}=-k_{hr}\cdot C_{r}$$

(4) $$\frac{dC_{m}}{dt}=-k_{hm}\cdot C_{m}$$

(5) $$\frac{dC_{s}}{dt}=-k_{hs}\cdot C_{s}$$

(6) $$\frac{dC_{aq}}{dt}=\left(k_{hr}\cdot C_{r}+k_{hm}\cdot C_{m}+k_{hs}\cdot C_{s}\right)-\left(k_{aq}\cdot C_{aq}\right)\;\;$$

(7) $$\frac{dC_{T}}{dt}=k_{aq}\cdot C_{aq}$$

where, *C_r_*, *C_m_* and *C_s_* are the fractions of the rapidly, moderately and slowly hydrolyzable organic solid carbon in the initial PLAMCB, respectively. *C_aq_* is the fraction of the mineralizable water-soluble intermediate carbon. Likewise, *k_hr_*, *k_hm_* and *k_hs_* are the hydrolysis rate constants of the rapidly, moderately and slowly hydrolyzable solid carbon fractions, respectively, and *k_aq_* the ultimate mineralization rate constant, describing the formation of CO_2_ from the water soluble intermediate. A lag phase C was included during the solving of rate equations, Equations (2)–(7), to account for the initial days of the process when first-order biodegradation kinetics does not apply [35]. The solutions of rate equations are shown in Equations (8)–(12)

(8) $$C_{r\_t}=C_{r0}\cdot e^{-k_{hr}\cdot \left(t-c\right)}$$

(9) $$C_{m\_t}=C_{m0}\cdot e^{-k_{hm}\cdot \left(t-c\right)}$$

(10) $$C_{s\_t}=C_{s0}\cdot e^{-k_{hs}\cdot \left(t-c\right)}$$

(11) $$C_{aq\_t}=C_{aq0}\cdot e^{-k_{aq}\cdot \left(t-c\right)}+C_{r0}\cdot k_{hr}\cdot \frac{\left(e^{-k_{hr}\cdot \left(t-c\right)}-e^{-k_{aq}\cdot \left(t-c\right)}\right)}{k_{aq}-k_{hr}}+C_{m0}\cdot k_{hm}\cdot \frac{\left(e^{-k_{hm}\cdot \left(t-c\right)}-e^{-k_{aq}\cdot \left(t-c\right)}\right)}{k_{aq}-k_{hm}}+C_{s0}\cdot k_{hs}\cdot \frac{\left(e^{-k_{hs}\cdot \left(t-c\right)}-e^{-k_{aq}\cdot \left(t-c\right)}\right)}{k_{aq}-k_{hs}}$$

(12) $$C_{Tt}=\left\{\begin{array}{c} C_{aq\_0}\cdot \left(1-e^{-k_{aq}\left(t-c\right)}\right)\\ +\left[C_{r\_0}\cdot (1-\frac{k_{aq}}{k_{aq}-k_{hr}}e^{-k_{hr}\left(t-c\right)}+\frac{k_{hr}}{k_{aq}-k_{hr}}e^{-k_{aq}\left(t-c\right)})\right]\\ \;\;\;\;+\left[C_{m\_0}\cdot (1-\frac{k_{aq}}{k_{aq}-k_{hm}}e^{-k_{hm}\left(t-c\right)}+\frac{k_{hm}}{k_{aq}-k_{hm}}e^{-k_{aq}\left(t-c\right)})\right]\\ +\left[C_{s\_0}\cdot (1-\frac{k_{aq}}{k_{aq}-k_{hs}}e^{-k_{hs}\left(t-c\right)}+\frac{k_{hs}}{k_{aq}-k_{hs}}e^{-k_{aq}\left(t-c\right)})\right] \end{array}\right\}$$

$$for\;t>c\;\;\;or\;C_{Tt}=0\;for\;t<0$$

where *C_ro_*, *C_mo_* and *C_so_* refer to the initial percentages of rapidly, moderately and slowly hydrolyzable solid carbon fractions, respectively, while *C_rt_*, *C_mt_* and *C_st_* are the percentages of the same fractions at time *t*. *C_aq0_* and *C_aqt_* are the percentage of the intermediate water soluble carbon at *t* = 0 and at *t* = *t*, respectively. Also, *C_Tt_* is the cumulative percentage of CO_2_ production.

All the kinetic parameters were constrained to be positive. Mathematically, the constraints were as follows:

(i) The initial total organic solid carbon *C_ti_* (%) comprises the initial readily, moderately, slowly and the initial percentage of intermediate soluble carbon:

*C_ti_* = *C_ro_* + *C_mo_* + *C_so_* + *C_aq0_* (13)

(ii) A similar equation can be established for final conditions:

*C_tf_* = *C_rf_* + *C_mf_* + *C_sf_* + *C_aqf_* (14)

(iii) For the rate constants:

*0*≤ *k_hs_* < *k_hm_* < *k_hr_* < <*k_haq_* (15)

(iv) Likewise, all the kinetic parameters have positive values.

## 3. Results and Discussion

### 3.1. Compost Characterization

The compost was sieved to a 10 mm average particle diameter, and its initial properties were: pH 7.92 ± 0.015, total solids: 49.75 ± 0.15%, humidity: 50.2 ± 0.2%, volatile solids: 42.06 ± 0.14%, organic carbon: 23.36 ± 0.08% and nitrogen: 1.56 ± 0.025%. According to the data obtained and research reported previously, the parameters showed that it corresponded to mature compost, which allows it to be suitable for use as a means of biodegradation [42,47].

### 3.2. Biocomposites Characterization

#### 3.2.1. Raman Spectroscopy

**Figure 2:**
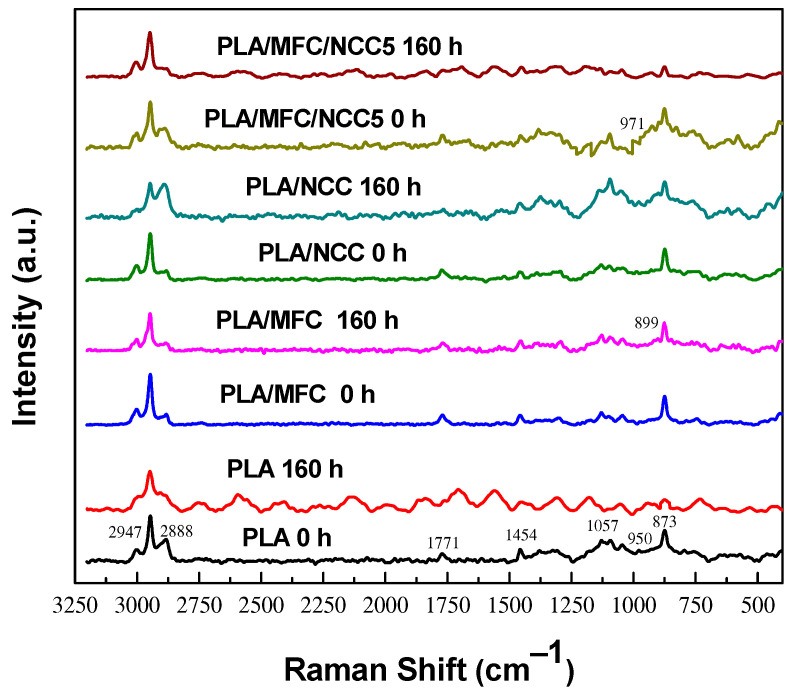
Raman spectra of PLA, PLA/MFC, PLA/NCC and PLA/MFC/NCC5 biocomposites before and after 160 h of thermo-oxidation at 70 °C.

The Raman spectra of the PLAMCBs, before and after thermo-oxidation at 70 °C for 160 h, can be seen in Figure 2. The peaks located at 2995 and 2970 cm^−1^ correspond to a CH_3_ asymmetric stretching bond, whereas the peak at 2946 cm^−1^ is due the symmetric one. The CH_3_ scissoring bend in plane can be seen at 1454 cm^−1^ and the C-O-C stretching at 1090 cm^−1^. The C=O stretching bond can be seen at 1771 cm^−1^. The peak at 955 cm^−1^ due to C-C stretching is related to the PLA amorphous phase. After thermo-oxidation, it can be seen that the peaks at 873 and 955 cm^−1^ decreased, indicating the oxidation of the PLA backbone chain and an increase in the crystalline phase [46]. Regarding the materials that contain cellulosic fibers, bands at 971 and 897 cm^−1^ can be seen which are characteristic of cellulose glycosidic bond stretching. Also, an increase in the width and height of peak at 2888 cm^−1^ can also be seen, assigned to cellulose CH and CH_2_ bonds, after thermo-oxidation at 70 °C. This behavior suggests that cellulosic fibers could be degraded during thermo-oxidation [8,45,48].

#### 3.2.2. Thermogravimetric Analysis (TGA)

**Figure 3:**
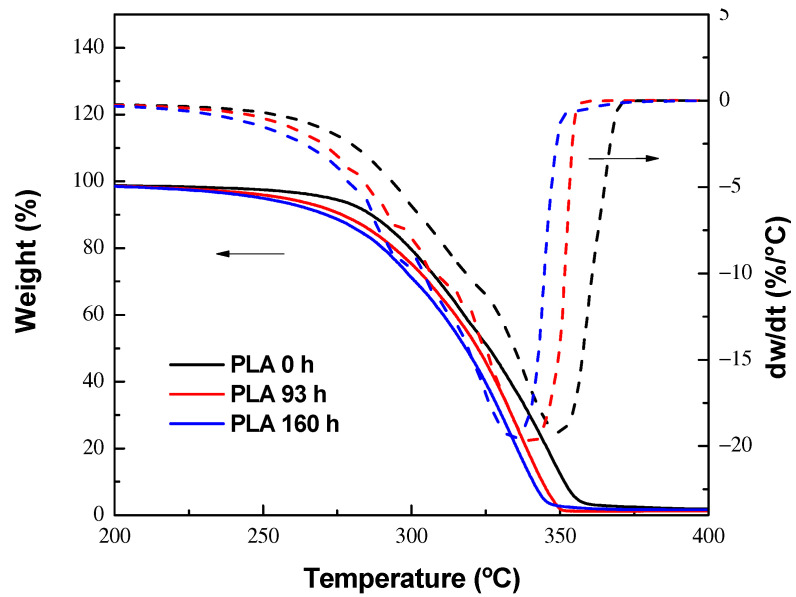
TGA and DTGA thermograms of thermo-oxidized PLA at 0, 93 and 160 h.

In Figure 3, the TGA and DTGA thermograms for PLA thermo-oxidized at different times are shown, where a 15 °C decrement in the degradation temperature of 160 h thermo-oxidized PLA was observed, compared to pristine PLA. This suggests that during the thermal oxidative treatment, in addition to its surface oxidation, there was also a drop in molecular weight [8,24,33,49,50].

**Figure 4:**
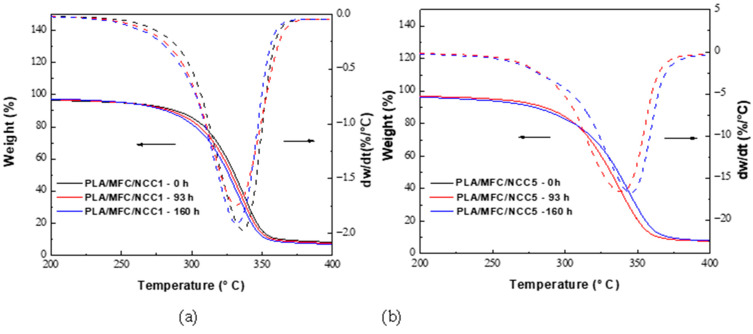
TGA and DTGA thermograms of thermo-oxidized biocomposites PLA/MFC/NCC for (**a**) NCC 1% and (**b**) NCC 5%.

Figure 4 shows the thermogram of thermo-oxidized multiscale composite materials compared with the pristine ones. From 250 °C, a decrease in weight begins in the samples; however, from 280 °C, an anticipated loss of weight is observed for the three samples. This premature loss is due to the treatments carried out in the manufacture of the composite material, as well as the thermo-oxidative treatment, and this would indicate the beginning of the decomposition of PLA and cellulose [8,45].

On the other hand, the composite materials presented a decrease in the degradation temperature as the NCC content increased, as did the thermo-oxidative treatment. This may be because by decreasing the molecular weight, the thermal stability also decreased, since it has been reported that when adding NCC to PLA, the thermal stability decreases due to the sulfate groups [51,52].

#### 3.2.3. Biodegradation Kinetics

**Figure 5:**
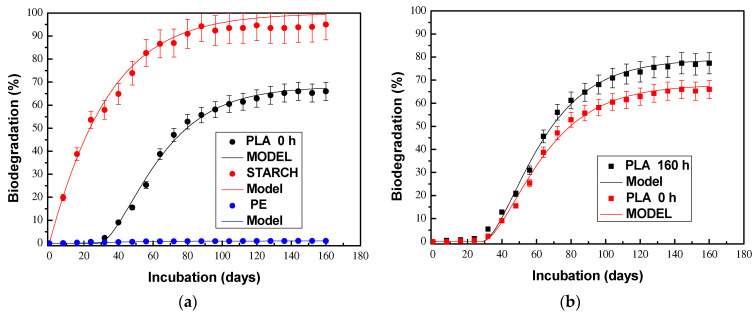
Biodegradation curves for: (**a**) positive (starch), negative (PE) controls and pristine PLA; (**b**) pristine and 160 h thermo-oxidized PLA.

To verify that the respirometric system complies with the requirements established in the ASTM D5338 standard, the biodegradation of pristine PLA and thermo-oxidized PLA was carried out using starch as a positive control and low-density polyethylene as a negative control. The results of these tests are presented in Figure 5, where it can be seen that the positive control (starch) degrades by more than 70% during the first 45 days, and there is no induction time or lag phase.

On the other hand, it is evident that the negative control (PE) practically does not biodegrade. Regarding the pristine PLA, it can be observed that it presents a typical sigmoidal-type biodegradation curve with a lag phase of almost one month and a maximum biodegradation of 65%. With regard to thermo-oxidized PLA, a similar behavior can be observed in terms of the evolution of biodegradation but with a shorter delay phase (25 days) and greater biodegradation (72%). These results show that the respirometric system is suitable for this study and that the abiotic treatment (thermo-oxidation) increases the biodegradability of PLA under controlled composting conditions [12,15,42,47].

**Figure 6:**
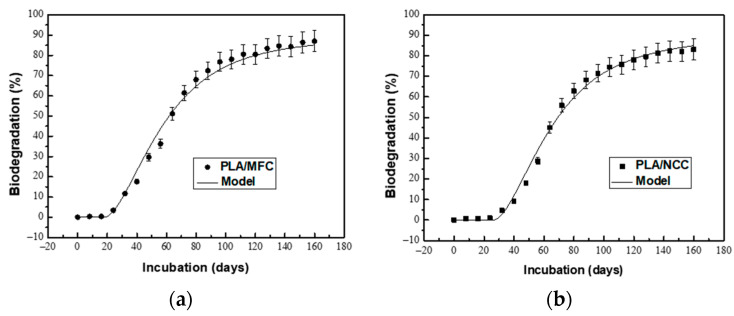
Biodegradation curves for (**a**) PLA/MFC and (**b**) PLA/NCC biocomposites.

Figure 6a shows the biodegradation curve for the PLA/MFC biocomposite material where the presence of an 18-day lag phase can be seen, and it can be observed that the material reached around 87% of mineralization after 180 days of incubation. Comparing these results with pristine PLA, it can be seen that the incorporation of 20% cellulose microfibers increased PLA biodegradation by 30%, whereas the lag phase decreased by −35%. Compared to thermo-oxidized PLA, the increase in mineralization and the decrease in lag phase were 13% and −25%, respectively. These findings are in agreement with those reported for several authors [18,19,20]. The biodegradation curve for PLA/NCC nanocomposite can be seen in Figure 6b, where it can be observed that an 83% biodegradation was reached after 180 days of composting, with a lag phase of 24 days. Compared with the thermo-oxidized PLA, the presence of 5% of cellulose nanocrystals enhances the PLA biodegradation in 8%, while the lag phase remains similar. Comparing PLA/NCC with PLA/MFC, it can be seen that both cellulosic fibers promote similar PLA biodegradation, although their contents are very dissimilar. This behavior may be due to the greater amount of contact area that the NCCs have with the PLA matrix due to its nanometer size [21].

**Figure 7:**
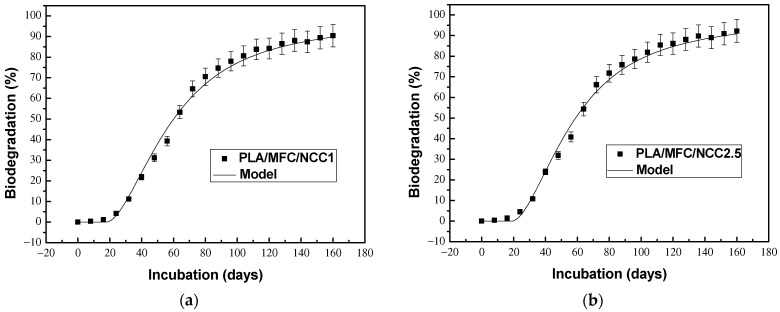

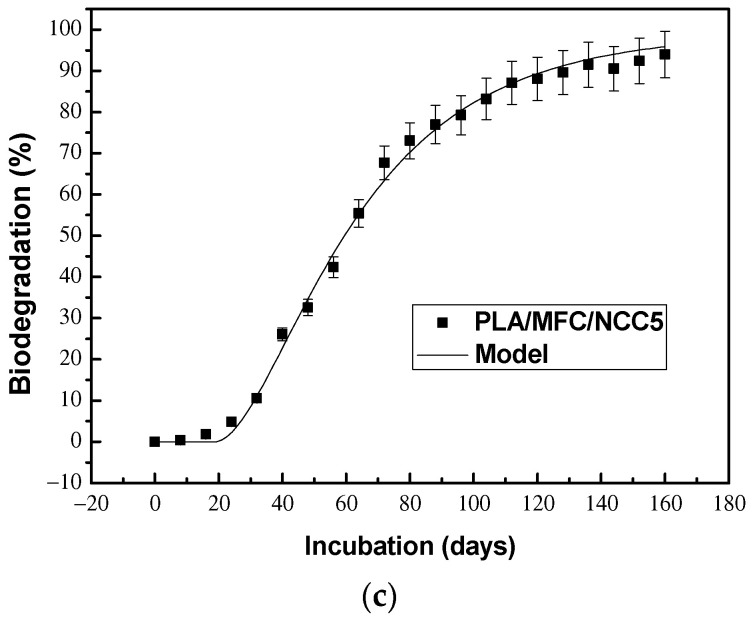
Biodegradation curves for the multiscale biocomposites (PLA/MFC/NCC) with different NCC contents: (**a**) 1%; (**b**) 2.5% and (**c**) 5%.

Figure 7a shows the biodegradation curve for the PLAMCB containing 1% cellulose nanocrystals, while maintaining a total of 20% cellulosic reinforcements, PLA/MFC/NCC1. In this figure it can be seen that the biodegradation reached 90% and the induction time or lag phase was 18 days. Compared with PLA/MFC, we can see that the substitution of 1% of the cellulose microfibers by cellulose nanocrystals does not modify the induction time but does increase the biodegradability of the composite material by 3%. Figure 7b shows the curve corresponding to PLA/MFC/NCC2.5, where it can be seen that the effect of substituting 2.5% of MFC for NCC on the biodegradation of the multiscale material was a 10% reduction in induction time and a 6% increase in mineralization, compared to PLA/MFC. Regarding the PLA/MFC/NCC5 material, its biodegradation curve is shown in Figure 7c. In this figure it can be seen that the mineralization of the multiscale composite material reached 94%, that is, an increase of 9% compared to PLA/MFC. In this case, the decrease in induction time was also 10%. These results show that the NCCs, in presence of cellulose microfibers, enhance the biodegradability of PLA and are in agreement with the results reported by other authors [21,22,23,24,25]. Galera-Manzano et al. [53] studied the drum-composting biodegradation of PLA multiscale composites and reported that a synergistic effect exists when both cellulosic fibers were incorporated simultaneously in the biocomposites.

#### 3.2.4. Kinetics Analysis

The experimental results of mineralization obtained during the biodegradation study under controlled composting conditions of the different materials were analyzed with the Leejarkpai et al. [35] model that includes the lag phase and which is described by Equation (12) and subject to the restrictions established in the Equations (13)–(15). To determine the values of the different parameters that appear in the model, three initial solid carbon fractions (*C_r0_*, *C_m0_*, *C_s0_*) and their respective hydrolysis kinetic constants (*k_hr_, k_hm_, k_hs_*), the initial soluble carbon fraction (*C_aq0_*) and its mineralization kinetic constant (*k_haq_*). Nonlinear parametric fitting was carried out using Origin^TM^ 8.5 software.

Table 2 shows the results of the adjusted parameters for each of the materials studied and their adjustment coefficient (R^2^). In this table it can be seen that the initial fraction of soluble carbon (*C_aq0_*) in PLAMCBs is four orders of magnitude higher compared to thermo-oxidized PLA. Regarding the easily hydrolyzable initial fraction (*C_r0_*), it can be seen that this fraction is below 10% for all studied materials. Also, it can be observed that the incorporation of cellulose microfibers increases its value 6 times with respect to PLA, and by substituting the MFC with 1 and 2.5% of NCC, *C_r0_* decreases by 67%. On the other hand, when NCC content is 5%, the *C_r0_* value decreases only 33%. This behavior can be attributed to NCC agglomeration in the biocomposites. It is evident in Table 2 that the *C_m0_* fraction predominates in all materials. It ranges from 66% for PLA/MFC/NCC5 to 77% for PLA. It can be seen that the incorporation of cellulosic reinforcements decreases this fraction by up to 15%, with nanocrystals promoting this decrease to a greater extent. These results corroborate the crystallinity slow-down PLA biodegradation that other authors have pointed out [14,25,27,50,51,53]. Regarding the initial fraction that is most recalcitrant to biodegradation (*C_s0_*), it can be seen that it ranges from 20 to 30% in the materials studied. The incorporation of MFC does not seem to modify it, compared to PLA, but it is evident that the NCC content increases it by 50%; that is, the replacement of microcellulose by nanocellulose has an appreciable impact on the initial amount of recalcitrant carbon in the materials during composting. This finding suggests the NCC promotes water diffusion to the biocomposites’ bulk [53].

Regarding the kinetic constants, it can be seen that the mineralization constant (*kaq*) adjusted for all the materials is the same, reflecting that there is no difference in the origin of the solubilized carbon in terms of mineralization; that is, once a solid carbon is solubilized, it loses its identity and is assimilated at the same rate by microorganisms. Regarding the kinetic constants of hydrolysis of the easily and moderately soluble fractions (*C_r0_* and *C_m0_*), it can be observed that at most they are 40% lower than the mineralization constant. Analyzing them separately, it is observed that the incorporation of MFC to PLA does not affect the rate of hydrolysis of the *C_r_* fraction of the composite material. On the other hand, the *C_r_* hydrolysis rate decreases by 16% with the incorporation of NCC, regardless of its quantity. As far as the fraction *C_m_* is concerned, the behavior is a bit more complex. The hydrolysis rate decreases by 30% with the incorporation of MFC. Substitution of MFC with 1 and 2.5% NCC makes hydrolysis 15% faster; however, with 5% NCC content in the multiscale composite, the hydrolysis rate of the *C_m_* fraction is similar to that of PLA/MFC; that is, it hydrolyzes at a similar rate when there is only cellulose microfibers.

From the *k_hs_* values reported in Table 2, it can be seen that the inclusion of cellulosic fibers in PLA favors that its most recalcitrant fraction is much more susceptible to hydrolysis. The incorporation of MFC favors the hydrolysis of *C_s_* at rates 2 orders of magnitude higher. When substituting MFC with NCC, this hydrolysis speed increases even more, reaching three orders of magnitude, with respect to PLA, when the content of substitution of MFC with NCC in the multiscale composite material reaches 5%. These results show that the presence of cellulose nanocrystals plays a very important role in the biodegradation of PLA present in this new generation of advanced multiscale biomaterials when composted under controlled conditions.

**Figure 8:**
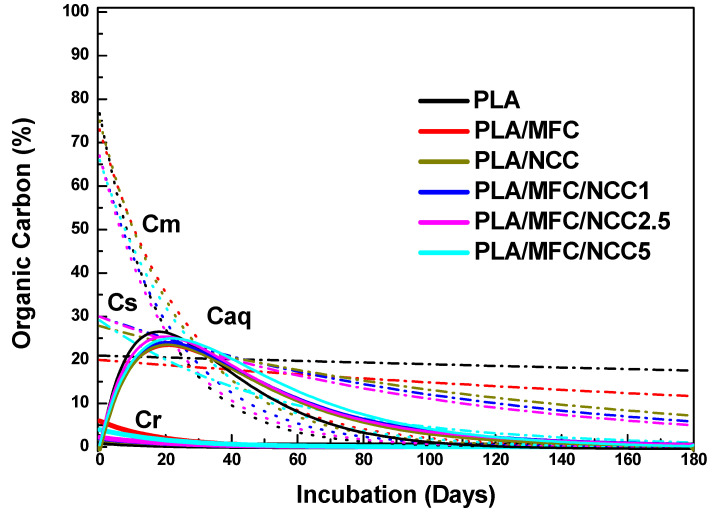
Curves of organic solid carbon fractions (Cr, Cs and Cs) and the organic carbon soluble fraction (Caq) with the incubation time for the different materials studied.

Figure 8 shows the evolution of the different carbon fractions present in the composite materials during incubation times under controlled conditions. In this figure, it can be seen, as expected, that the easy, moderate and slowly hydrolyzable solid carbon fractions show a decay behavior, since during the entire composting process these fractions become solubilized as they hydrolyze.

On the other hand, the fraction of carbon soluble in water presents a very different behavior, similar to a logarithmic distribution, since it starts with low values that increase with time, reaches a maximum and then experiences a decrease.

This behavior is due to the fact that once the induction time is exceeded, the amount of carbon that is solubilized is greater than the amount that is mineralized by the microorganisms and therefore grows over time. As the amount of carbon that is solubilized due to the depletion of the solid carbon fractions present in the compost, the amount of carbon that is mineralized exceeds that which is solubilized, and therefore the *C_aq_* fraction present in the compost begins to decline over time.

**Figure 9:**
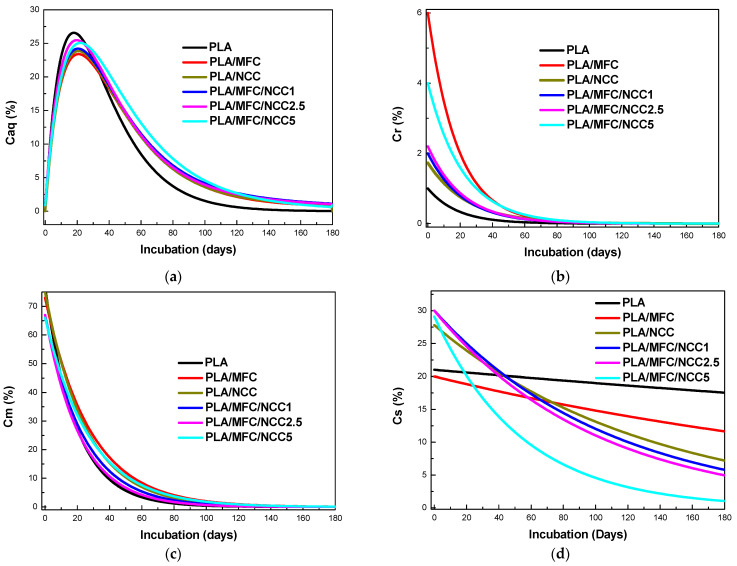
Evolution of organic carbon fractions present in the different materials studied with incubation time: (**a**) Aqueous soluble organic fraction (*C_aq_*); (**b**) readily (*C_r_*); (**c**) moderately (*C_m_*); and (**d**) slowly (*C_s_*) hydrolyzable solid organic fractions.

The individual behaviors of the different carbon fractions present in the multiscale materials studied as a function of incubation time are presented in Figure 9.

In Figure 9a, it can be seen that the maximum concentration of *C_aq_* occurs around 30 days after the biodegradation process begins, that is, once the induction time elapses. The highest value of *C_aq_* is obtained for PLA and the lowest for PLA/MFC. It can be seen that the replacement of MFC with NCC gradually increases the maximum value reached by *C_aq_*, although without reaching the value of PLA. Likewise, it is seen that the *C_aq_* fall follows an exponential behavior much faster for PLA than for multiscale composite materials. The *C_aq_* drop in these materials is very similar and does not depend on the content of cellulose nanocrystals.

In Figure 9b, it can be seen that the exponential drop in Cr is lower for PLA and much higher for PLA/MFC and that, as the presence of NCC increases in multiscale materials, the speed with which the fraction of *C_r_* disappears, that is, becomes solubilized, decreases, although it is always longer than that of PLA.

In the case of the moderately hydrolyzable solid carbon fraction, it is evident that Cm follows an exponential decline for all materials, and the rate of decline is higher for PLA/MFC and lower for PLA, as observed in Figure 9c. So, the effect of replacing MFC with NCC is a gradual decrease in the rate of disappearance of *C_m_* as NCC increases. However, this effect is less impressive than with *C_r_*.

It is evident that the behavior of the most recalcitrant fraction, *C_s_*, unlike the aforementioned fractions, presents two different regimes of disappearance for the studied materials, as can be seen in Figure 9d. A linear fall is appreciated, with different slopes, for the PLA and for PLA/MFC. On the other hand, for multiscale materials containing cellulose nanocrystals, the disappearance of *C_s_* follows an exponential behavior, whose rate of decline depends on the content of NCC, being much faster with NCC5. If we examine the residual organic carbon content for each material, after 180 days of incubation, we see that for PLA it is around 20% and for NCC0 12%. In the case of multiscale materials, the residual Cs is 8.6 and 3% for NCC1, NCC2.5 and NCC5.

It is known that the hydrolysis of PLA consists of two stages. In the first of which, there is a drop in molecular weight and an increase in the percentage of crystallinity, without weight loss. When a critical molecular weight value is reached, the second stage begins, which consists of the solubilization of the oligomers formed and therefore weight loss. This is essentially a surface erosion process [54,55,56,57]. However, the results found suggest that the incorporation of cellulose nanocrystals in the PLA/MFC composite facilitates the entry of water into the material and therefore promotes hydrolytic degradation from the inside and not only by erosion, promoting the solubilization of the oligomers formed that then are metabolized by the microorganisms. That is why the hydrolysis constant of the recalcitrant material of the PLAMCBs is greater in the presence of the NCC, and the drop in *C_s0_* is exponential.

## 4. Conclusions

In this work, the study of the kinetics of biodegradation of multiscale composite materials of PLA, microcellulose (MFC) and cellulose nanocrystals (NCCs) was carried out under controlled composting conditions according to the recommendations established in the ASTM D-5338 standard.

A first-order model, based on Monod’s kinetics under limiting substrate conditions, was used to study the effect of cellulose nanocrystal (NCC) content on the biodegradation kinetics of multiscale composite materials (PLAMCBs). It was found that the incorporation of cellulosic fibrous reinforcements increases the biodegradability of PLA by promoting hydrolysis during the first stage of composting. Likewise, it was found that the partial substitution of microcellulose (MFC) by cellulose nanocrystals (NCCs) increases the biodegradability of the multiscale biocomposites. This increase was more evident as the NCC content increased, and it was attributed to the fact that the incorporation of cellulose nanocrystals facilitates the entry of water into the material and therefore promotes the hydrolytic degradation of the most recalcitrant fraction of PLA from the bulk and, not only by surface erosion, enhancing the solubilization of the oligomers formed that then are metabolized by the microorganisms.

Also, it was found that the thermo-oxidation of multiscale composite materials at 70 °C for 160 h increases the biodegradability of PLA and multiscale composite materials.

## Figures and Tables

**Table 1:** PLAMCB formulations used in this study.

Material	Cellulose Microfiber Content (%)	Cellulose Nanocrystal Content (%)
PLA	0	0
PLA/MFC	20	0
PLA/NCC	0	5
PLA/MFC/NCC1	19	1
PLA/MFC/NCC2.5	17.5	2.5
PLA/MFC/NCC5	15	5

**Table 2:** Kinetic model parameters and coefficients of determination (R^2^).

Material	*C_aq0_* (%)	*k_aq_* (day^−1^)	*C_r0_* (%)	*k_hr_* (day^−1^)	*C_m0_* (%)	*k_hm_* (day^−1^)	*C_s0_* (%)	*k_hs_* (day^−1^)	c (days)	R^2^
PLA	0.0001	0.06	1.0	0.055	76.7	0.052	21	1 × 10^−5^	24	0.991
PLA/MFC	1.0	0.06	6	0.055	73	0.036	20	0.003	18	0.991
PLA/NCC	0.01	0.06	1.8	0.041	75	0.039	24.2	0.007	22	0.990
PLA/MFC/NCC1	1.0	0.06	2	0.046	67	0.042	30	0.0091	18	0.999
PLA/MFC/NCC2.5	1.0	0.06	2	0.046	67	0.042	30	0.01	16	0.991
PLA/MFC/NCC5	0.9	0.06	4	0.046	66	0.036	29.1	0.0185	16	0.991

## Data Availability

The data presented in this study are not available since they are part of an ongoing study.
